# Attitude of Jordanian Physicians toward Biochemistry and Genetics

**DOI:** 10.1155/2019/3176951

**Published:** 2019-11-13

**Authors:** Mahmoud A. Alfaqih, Yousef S. Khader, Nabil Bashir, Zuhair Nusair, Quteiba Nuseir, Mohamad Nusier

**Affiliations:** ^1^Department of Physiology and Biochemistry, Jordan University of Science and Technology, Irbid 22110, Jordan; ^2^Department of Community Medicine and Public Health, Jordan University of Science and Technology, Irbid 22110, Jordan; ^3^Irbid Military Hospital, Royal Medical Services, Irbid, Jordan

## Abstract

**Background:**

Several studies found that physicians develop a negative attitude toward biochemistry and genetics disciplines. Many medical schools adopt an integrated system-based curriculum supplemented with clinical correlations. Medical schools in Jordan switched to the integrated curriculum; however, studies that evaluate the attitude of physicians toward biochemistry and genetics are lacking.

**Objectives:**

This study aimed to evaluate the attitude of physicians toward biochemistry and genetics including the correlation of their curricula with clinical practice.

**Materials and Methods:**

A structured questionnaire consisting of 40 statements was distributed to a random sample of 616 physicians practicing in private and governmental hospitals in Jordan. Participants earned their MD or MBBS degree from Jordan or other countries and were interns, residents, or specialists.

**Results:**

More than half of the participants admitted that biochemistry and genetics are intellectually challenging and were among their least favourite subjects (59.1%); however, many of them were familiar with some of the contemporary advances in biochemistry and genetics and their translational potential (64.0%). Most of the participants felt that modifying the medical school curriculum by integrating biochemical and genetic concepts with clinical teaching will motivate the medical students (74%). In univariate analysis, residents showed the most positive attitudes and were the most knowledgeable about the biochemical changes associated with diseases and about the contemporary advances in biochemistry or genetics (*P* < 0.05). In multivariate analysis, physicians practicing in the private sector or those with more than five years of experience generally had a more positive attitude toward biochemistry and genetics (*P* < 0.05).

**Conclusion:**

Physicians in Jordan showed an overall positive attitude toward biochemistry and genetics. This was more evident among residents, physicians with more than five years of experience, or those practicing in the private sector.

## 1. Introduction

Biochemistry and genetics are an integral part of the medical school curriculum [1]. These basic medical sciences attempt to explain the activity of living systems at the molecular level including an understanding of various metabolic and signalling pathways operating within the cells during different environmental and health/disease conditions [1]. Such an understanding is essential for elucidating the underlying mechanisms of multiple disease processes. Furthermore, this knowledge could be utilized for the development of new therapeutic interventions and the discovery of novel biomarkers that aid in the early detection of diseases and/or monitoring their progression [2]. Moreover, with the introduction of next-generation sequencing and the recent advances in gene therapy, these two fields are already playing an even more important role in modern medicine [3]. Indeed, previously abstract concepts like personalized medicine and pharmacogenetics are becoming a part of routine clinical practice [4].

Unfortunately, despite the importance of having a solid foundation in biochemistry and genetics for clinical practice, multiple studies demonstrated that a significant proportion of general medical practitioners have deficiencies in their knowledge on the above disciplines [5–7]. Multiple reasons were put forward to explain this observation: (a) the tedious and unengaging instruction methodology adopted in medical schools [8], (b) the biochemistry and genetics syllabi which are saturated with factual data and abstract concepts [9], and (c) the incorrect notion held by many medical school graduates that the concepts taught in biochemistry and genetics are irrelevant to medical practice [10]. Collectively, all these lead sometimes to a negative attitude toward biochemistry and genetics disciplines among medical school graduates. Interestingly, however, it appears that the negative perception among physicians changes after obtaining their first medical degree; indeed, some studies showed that physicians gain more interest toward biochemistry and genetics after graduation [11, 12]. This is, however, accompanied by a general feeling that their knowledge base in biochemistry and genetics could have been more adequate [11]. This finding lends support to the argument that the attitude of physicians toward biochemistry and genetics could become more positive if the teaching methods of biochemistry and genetics have a better correlation with the following clinical teaching years and postgraduation practice/research [13].

In Jordan, a mandatory degree in premed school is not a prerequisite for admission. Medical students start their 6-year-long degree directly after secondary school [14, 15]. In the medical school curriculum, the core concepts of biochemistry and genetics are introduced during the first and second years as independent subjects [14, 15]. However, during the second semester of the second year and the entire third year, these subjects are taught as part of a modular system-based curriculum where each module covers only one organ system. Notably, this curriculum has only been implemented since 1999, and before that date, the biochemistry and genetics courses were solely taught as independent subjects over the course of the first three years [14, 15]. This entails that physicians currently practicing in Jordan have received their biochemistry and genetics courses using either the older traditional curriculum or the more recent modular-based syllabus. Additionally, current estimates indicate that around 20% of physicians in Jordan have received their MD or MBBS degree from foreign medical schools [16]. Given the above profile, physicians in Jordan represent a unique blend exposed to different teaching styles in basic medical sciences depending on the year, or country of their graduation with an MD or MBBS degree.

Despite the differences explained above in how Jordanian physicians received their biochemistry and genetics courses and the known dichotomy in how physicians perceive these disciplines, there are no studies that investigate the attitude of Jordanian physicians toward these fields. Given the above deficiency, this study aimed to assess the knowledge and the attitude of physicians of various professional levels and backgrounds toward the contemporary advances in biochemistry and genetics. This study also aimed to evaluate the content of the biochemistry and genetics curricula offered in Jordanian medical schools including their correlation with clinical practice.

## 2. Materials and Methods

### 2.1. Study Design

This study is cross-sectional in design. Following appropriate ethical approvals and funding from the Deanship of Research at JUST (368/2016), the Jordan Medical Association database was queried for interns (physicians enrolled in a mandatory one-year training program following their graduation) and for residents and specialists from each of the following disciplines: gynaecology, paediatrics, endocrinology, and internal medicine. Using the above database, a simple random sample of 710 physicians (interns, residents, and specialists) practicing in northern, central, and southern Jordan was identified by their ID registration numbers. From this list, 616 physicians were invited to participate in this study based on the following criteria: (a) obtaining the first medical degree (MD or MBBS) between the years of 1985 and 2016 and (b) not sitting for advanced biochemistry or genetics (molecular, biochemical, or cytogenetics) training following graduation including graduate degrees (diplomas, MSc, and PhD), board certifications, or continuing medical education (CME) courses of more than six credit hours. To complete the survey, participants were interviewed face to face by two residents from the public health residency program at King Abdullah University Hospital (KAUH), Irbid, during the period from January to July 2016. Prior to completing the survey, the purpose of the study was explained to the participants who then signed a consent form attached to the frontal page of the survey. Five hundred and fourteen physicians (response rate 83%) completed the survey. The invited physicians who did not complete the survey listed their fully occupied schedule or a general lack of interest in the topic as their main reasons. Unfortunately, most physicians who did not complete the survey were from Southern Jordan; we thus excluded this geographic location from the final analysis (Table 1).

### 2.2. Study Questionnaire

A structured questionnaire was designed to include 40 statements. The questionnaire was divided into four sections. The four sections were intended to assess the physician's attitude and viewpoints toward the following areas: (a) the integration of biochemistry and genetics concepts into clinical practice (Table 2), (b) contemporary advances in molecular medicine (Table 3), (c) the need for continuous education in biochemistry and genetics (Table 4), and (d) the current level of knowledge on biochemistry and genetics among the medical students (Table 5). Questions in each section were drafted by graduate students and were later modified by three faculty members of the Department of Physiology and Biochemistry at Jordan University of Science and Technology each in their area of expertise. The survey was reviewed and modified for clarity, content, and ethical considerations by a senior statistician. A pilot study on 82 subjects was first carried out at KAUH to test the survey methods, and to assess the clarity of the questionnaire. Based on the pilot study, the questionnaire was revised accordingly.

### 2.3. Data Collection and Analysis

The data were analysed using IBM-Statistical Package for Social Sciences (IBM-SPSS) version 22 (Armonk, NY). All variables in the study were categorical. The participants were first grouped according to their practice location (central or northern), their employment sector (private or public), or the country that awarded the MD or MBBS degree (Jordan or other countries) and were then cross-tabulated with their title (intern, resident, or specialist). The frequency of participants who answered yes or no to each question was calculated. Pearson's chi-square test was used to test for statistically significant differences in the participants' response according to each of the following four variables: practice location, employment sector, country of MD or MBBS, and title. To perform multivariate analysis, the response of participants to each statement was assessed using a 5-point Likert scale. An average attitude score was then calculated for each domain by calculating the mean of all responses (ranging from 1 to 5) to all the statements listed in that specific domain. A higher score indicated more positive attitude. Multivariate analysis of variables affecting the mean score of each domain was conducted using a general linear procedure (GLP). A *P* value less than 0.05 was considered statistically significant.

## 3. Results

### 3.1. Sample Population

The response rate to the survey was 83%. Many of the interns (84.2%), residents (76.6%), and specialists (68.5%) who completed the survey were practicing in the private and public sectors in the northern part of Jordan at the time of the interview (Table 1). Furthermore, most of the interns (90.3%), residents (88.7%), and specialists (69.4%) obtained their first medical degree (MD or MBBS) from Jordan (Table 1). All the interns (100%) were practicing in hospitals affiliated with the public health sector. Moreover, majority of the residents (82.1%) and specialists (97.3%) were practicing in the public health sector as well. About three quarters of the interns (70.9%) and residents (72.4%), who were practicing in the public health sector, were working in hospitals affiliated with the Ministry of Health, while most of the specialists practicing in the public sector were working in hospitals affiliated with the Royal Medical Services (81.7%) at the time of the interview (Table 1).

In univariate analysis, only minimal variation was observed in the response of the study participants to the statements of the survey when comparisons were made according to the location of their employment (northern or central), country where they obtained their MD or MBBS degree (Jordan or others), or employment sector (private or public) (data not shown). However, more variation was observed in the responses when participants were grouped according to their professional title (intern, resident, or specialist) at the time of the interview (Tables 2–5). In this case, statistically significant differences were observed in the response of the participants to 11 different statements (Tables 2–5). We will thus initially present our findings considering differences in the response of the participants following their stratification according to their job title.

### 3.2. Attitude of Sampled Physicians toward the Integration of Biochemistry and Genetics Concepts into Clinical Practice

Most of the sampled participants indicated that they employ biochemical and genetic tests to reach the correct diagnosis (89.9%) and appreciate the importance of biochemical and genetic markers in detecting the early stages of the disease (89.3%) (Table 2). Moreover, four out of five participants (80.6%) believed that the physical signs of certain diseases are preceded by subtle biochemical changes and that a comprehensive treatment strategy should ideally correct the molecular basis of diseases rather than solely managing the resulting signs and symptoms (81.7%). However, despite their appreciation of the necessity of having a solid background in biochemistry and genetics for modern clinical practice (75.3%), less than half of the participants (42.5%) indicated that they consult with a clinical biochemist on a regular basis to better assist them in clinical decision-making (Table 2).

A higher percentage of residents, than interns or specialists, indicated that they were knowledgeable about the biochemical changes that result from hormonal imbalance (85.2%, *P*=0.045) or acidosis and alkalosis (79.8%, *P*=0.022) (Table 2). Residents were also more in favour of a role for proper nutrition in disease prevention than interns or specialists (86.1%, *P*=0.011) (Table 2).

### 3.3. Attitude of Sampled Physicians toward Contemporary Advances in Molecular Medicine

Most participants were familiar with the role of biochemistry or genetics in designing better targeted therapies (64.2%) and the role of genetic polymorphisms in determining disease susceptibility (85.0%) (Table 3). However, participants were less familiar with some of the breakthroughs in genetics like publishing the full sequence of the human genome (51.9%) and with concepts that recently gained more attention in the scientific community such as whole genome association studies (57.8%) and “epigenetics” (52.2%) (Table 3). Despite the positive attitude described above, there was a disparity among the participants in their support of establishing a DNA sequence database to expedite personalized medicine:74.4% of the residents supported this step, while only 64.8% of the specialists and 59.7% of the interns found this necessary (Table 3).

### 3.4. Attitude of Sampled Physicians toward the Need for Continuous Education in Biochemistry and Genetics

More than half of the interns (55.4%) and specialists (52.1%) admitted to not being familiar with the most recent molecular advances in their respective area of specialty/interest (Table 4). This may present a major hindrance discouraging them from reading relevant research articles where only 47.5% of the interns and 41.7% of the specialists feel that they have enough background in biochemistry and genetics to allow them to understand research articles that heavily depend on molecular-based techniques to support the findings (Table 4). However, a majority of the interns (63.9%) and specialists (75.3%) showed interest in attending CME courses, perhaps to address these and other deficiencies (Table 4). Noteworthy was the finding that the residents were significantly more familiar with the most recent molecular advances in their field (60.7%, *P*=0.003) and were significantly more open to enrollment in CME courses (80.5%, *P*=0.001) (Table 4).

### 3.5. Attitude of Sampled Physicians toward the Current Level of Knowledge on Biochemistry and Genetics among Medical Students

Most of the sampled participants believed that the medical school curriculum is still lacking in areas of biochemistry and genetics (63.6%) (Table 5). Moreover, more than two-thirds of the participants believed that biochemistry and genetics are intellectually challenging topics (68.9%) and were among their least favourite subjects of the medical school curriculum (59.1%) (Table 5). Given the above perception of the participants, it is not surprising that many of the participants recommended further restructuring of the curriculum to more efficiently integrate relevant clinical scenarios applicable to future bedside teaching (71.0%) (Table 5).

### 3.6. Multivariate Analysis of Variables Affecting Attitude of Physicians toward Biochemistry and Genetics

The results of this analysis are displayed in Table 6. Herein, the participants were first stratified according to their location, country of graduation, job title, employment sector, and years of experience. An attitude score (for each domain) was then calculated based on the response of the participants to each of the statements listed under each specific domain. A GLP was then used to test if significant differences in the attitude score were observed between different stratifications of the participants for each of the domains.

In this analysis, it was observed that the employment sector and years of experience were the only two variables significantly associated with any of the attitude scores of different domains included in the study questionnaire (Table 6). Specifically, physicians in the private sector had a significantly more positive attitude toward integration of biochemistry and genetics concepts into clinical practice (*P*=0.002), toward contemporary advances in molecular medicine (*P*=0.049), and toward the need for continuous education in biochemistry and genetics (*P* < 0.001). On the contrary, physicians with higher years of experience (>5 years) had a significantly more positive attitude toward integration of biochemistry and genetics concepts into clinical practice (*P*=0.007).

## 4. Discussion

The results of this survey depict the attitude of physicians from various educational and professional backgrounds toward the content of the biochemistry and genetics curricula and their correlation with clinical teaching and practice in Jordan. Conclusions drawn from this study are expected to better inform medical educators and health policy-makers of the deficiencies of the current teaching methods of biochemistry and genetics and to provide guidelines of the best approach to mend these deficiencies. Notable was the finding that more than half of the physicians were familiar with some of the contemporary advances in biochemistry and genetics and the impact they have on modern medicine. Indeed, physicians were familiar with concepts like personalized medicine, whole genome association studies, and pharmacogenetics. Our results also demonstrated that most physicians appreciate the role biochemistry and genetics play in modern clinical practice including disease diagnosis and targeted therapy. However, despite the overall positive attitude, physicians were still reluctant to consult a clinical biochemist to help in the diagnosis and/or in monitoring disease progression. This might be explained by (a) the small number of individuals that hold professional board certification in clinical biochemistry in Jordan and (b) the lack of national board certification programs/fellowships in clinical biochemistry. Accordingly, Jordanian physicians are not used to having clinical biochemists as members of their medical teams.

Despite the relatively high response rate of this survey (83%), most of the nonresponders were from the southern part of Jordan. The authors, therefore, opted to exclude this geographic location from the final analysis. Southern Jordan primarily depends on the public sector to provide health services, and all of the physicians initially selected to participate in the survey were affiliated with the public sector. Unfortunately, southern Jordan is an underserved area, and physicians observe a high bulk of patients on a daily basis; this might explain why most of the physicians from southern Jordan declined to complete the survey.

All of the medical schools in Jordan currently teach biochemistry and genetics core concepts initially as independent subjects and then as part of an integrated and multidisciplinary syllabus [14, 15]. Before the year 1999, biochemistry and genetics were solely taught as independent subjects [14, 15]. Nonetheless, most of the survey participants believed that biochemistry and genetics are still inadequately covered by the current medical school curriculum. Indeed, despite the switch to the modular-based syllabus described above, most of the participants felt that the current curriculum would still benefit from further integration of clinical-based scenarios. This result is in agreement with several previous studies in other countries [5, 17–20] and could be explained by the fact that, in many of the Jordanian medical schools, the change in the content of the syllabus was not accompanied by a concurrent change in the instruction style of the basic medical science courses. Indeed, the teaching style of these courses still relies heavily on didactic sessions and lacks active student engagement.

In univariate analysis and upon the stratification of study participants according to their job title, residents showed the most positive attitudes relative to interns and specialists and were generally more knowledgeable about the biochemical aspects of multiple pathophysiological processes such as acid-base homeostasis and hormonal imbalance. Residents were also more open to sitting for CME courses. The overall more positive attitude of residents toward biochemistry and genetics could be explained by the fact that residents are more settled and oriented in their respective career paths compared to interns [21]. Additionally, in view of the competitive nature of their position, residents are required to regularly follow recent updates in their field of interest/future specialty [22].

In multivariate analysis, it was observed that physicians practicing in the private sector generally have a more positive attitude toward biochemistry and genetics than physicians practicing in the public sector. This was most evident in the attitude of physicians toward their need for continuous education in biochemistry and genetics. The reason behind this difference in attitude is not clear but could be related to a heavier workload on physicians in the public sector and a general lack of incentive for a continued education in biochemistry and genetics. Regardless, if this finding is confirmed in larger studies, it would provide health policy-makers with a clear incentive to target physicians in the public sector with educational programs to change their attitude toward biochemistry and genetics especially that rapid advancement in biochemistry and genetics is transforming medical practice and research.

This study has several limitations: Although the overall response rate of the survey was considerably in the higher range (83%) of comparable surveys, most of the nonresponders were physicians practicing in hospitals located in southern Jordan (data not shown). The responses of the above physicians were excluded from the final analysis. Accordingly, the study does not represent the viewpoints of all physicians practicing in Jordan. A more comprehensive national-scale study is required to overcome this limitation. Moreover, the questionnaire provided a subjective view of how physicians rate themselves and did not objectively test how much knowledge they possess on genetics and biochemistry. For example, the wording of some of the statements listed in Table 2 favours an affirmative answer from the responders. Despite these limitations, this survey is the first of its kind in Jordan and should be informative to medical educators in view of its structure and sample size.

## 5. Conclusions

In conclusion, physicians in Jordan, especially the residents, showed an overall positive attitude toward biochemistry and genetics. However, they felt that further restructuring of the medical school curriculum is warranted to better engage the students in learning biochemistry and genetics. This process should involve adopting a more interactive approach that requires student engagement in problem-solving, and the early introduction of clinical-based scenarios before bedside teaching at the hospital. Moreover, a better appreciation of the role of clinical biochemists in clinical decision-making could be achieved through the development of national programs that offer board certification in biochemistry and genetics. In multivariate analysis, this study showed that physicians practicing in the private sector or those who have more than five years of experience have a more positive attitude toward biochemistry and genetics. Accordingly, physicians practicing in the public sector or those at the beginning of their career could be a good target of CME courses in biochemistry and genetics than other groups of physicians.

## Figures and Tables

**Table 1:** Sociodemographic characteristics of the participants.

	Interns, *N* (%)	Residents, *N* (%)	Specialists, *N* (%)
*Location*			
Northern	139 (84.2)	210 (76.6)	50 (68.5)
Central	26 (15.8)	64 (23.4)	23 (31.5)

*Country of MD or MBBS*			
Jordan	149 (90.3)	243 (88.7)	50 (69.4)
Others	16 (9.7)	31 (11.3)	22 (30.6)

*Employment sector*			
Private	0 (0.0)	49 (17.9)	2 (2.7)
Public	165 (100.0)	225 (82.1)	71 (97.3)

*Public hospital affiliation*			
Royal Medical Services	48 (29.1)	62 (27.6)	58 (81.7)
Ministry of Health	117 (70.9)	163 (72.4)	13 (18.3)

Data are presented as numbers and percentages.

**Table 2:** Attitudes and perceptions of Jordanian physicians toward the integration of biochemistry and genetics into clinical practice.

Statement	Interns (agree)		Residents (agree)		Specialists (agree)		Total		*P* value
	*N*	%	*N*	%	*N*	%	*N*	%	
I employ different biochemical and genetic tests to help reach the correct diagnosis and in following up the patients	145	89.0	247	90.5	64	88.9	456	89.8	0.291
I understand how important biochemical and genetic markers are in detecting the early stages of certain diseases including cancer, hepatic and renal dysfunction, and myocardial infarction	144	87.3	250	91.2	63	86.3	457	89.3	0.287
I understand that subtle biochemical changes precede, in most cases, the physical signs of certain diseases	136	84.0	212	77.9	60	82.2	408	80.6	0.182
I constantly emphasize to my patients the importance of a healthy diet comprising balanced fractions of macro- and micronutrients (including vitamins and trace minerals)	129	79.6	235	86.1	59	80.8	423	83.4	0.011
I am knowledgeable about the biochemical changes that result from hormonal imbalance	128	78.5	231	85.2	54	74.0	413	81.7	0.045
A comprehensive treatment strategy should ideally be directed toward correcting the molecular basis of the disease, if possible, rather than the sole management of the resulting signs and symptoms	120	74.1	228	84.1	64	87.7	412	81.7	0.022
I am familiar with some of the acidosis and alkalosis disease conditions that are best understood and managed in the relevant biochemical context	111	68.1	217	79.8	53	72.6	381	75.4	0.022
I consult with a clinical biochemist on a regular basis to reach my diagnosis and to follow up the patients after they receive treatment	65	40.4	120	44.6	28	38.4	213	42.5	0.69
I regularly apply biochemically derived themes and equations in my practice including formulas applicable to drug dosing, drug clearance, therapeutic drug monitoring, and detection of disease-specific biomarkers	99	61.9	180	66.4	46	63.9	325	64.7	0.629
Many of the procedures performed in medical practice require knowledge on biochemistry and genetics including sample collection, handling of specimens, and interpretation of lab results	130	81.3	222	81.3	59	80.8	411	81.2	0.995
I can efficiently interpret biochemical and genetic lab results and relate them to the overall clinical picture of the patient	123	78.8	221	81.0	53	73.6	397	79.3	0.389
I understand that it is essential to treat patients on an individual basis taking into consideration their genetic and environmental backgrounds	131	82.4	224	83.0	59	80.8	414	82.5	0.912

Data are presented as numbers and percentages. *P* values were calculated using the chi-square test.

**Table 3:** Attitudes and perceptions of Jordanian physicians toward the contemporary advances in molecular medicine.

Statement	Interns (agree)		Residents (agree)		Specialists (agree)		Total		*P* value
	*N*	%	*N*	%	*N*	%	*N*	%	
I appreciate the role played by biochemists in designing targeted therapy	103	65.6	169	62.8	47	66.2	319	64.2	0.787
Genetic or biochemical changes have been linked to many of the most common disorders including neuropsychiatric, autoimmune, endocrine, cardiovascular, and chronic inflammatory disorders	122	76.7	220	81.5	62	84.9	404	80.6	0.284
Genetic polymorphisms account for the patient's variation in susceptibility to disease development, its complications, and response to treatment	133	83.6	236	87.1	57	79.2	426	85.0	0.218
I believe that sequencing the entire human genome will affect the quality of health care worldwide	121	76.1	205	75.9	51	69.9	377	75.2	0.475
I am familiar with whole genome association studies and appreciate how these studies advanced our understanding of multifactorial diseases	83	52.2	168	62.2	37	50.7	288	57.8	0.059
I support the routine use of modern molecular techniques in primary prevention, early diagnosis, and long-term follow-up of patients including DNA sequencing and expression microarrays	103	65.2	177	65.6	44	61.1	324	64.9	0.776
I am familiar with the term “epigenetics” and I understand how molecular alterations at this level may contribute to health and disease	69	43.9	152	57.1	34	47.2	255	52.2	0.023
I support establishing a DNA sequence database as part of a national health care plan	95	59.7	201	74.4	46	64.8	342	69.0	0.005
I am aware that the sequence of the human genome was published in 2003	80	50.3	148	54.6	32	43.8	260	51.9	0.24
I believe that the concept of “personalized medicine” as a treatment strategy can be applied in the conceivable future	82	51.6	168	62.2	44	61.1	294	59.1	0.087
I believe that “pharmacogenomics” may have the potential to maximize the therapeutic gain obtained from drugs and minimize their untoward effects	111	69.8	206	76.9	56	76.7	373	74.8	0.244

Data are presented as numbers and percentages. *P* values were calculated using the chi-square test.

**Table 4:** Attitudes and perceptions of Jordanian physicians toward the need for continuous education in biochemistry and genetics.

Statement	Interns (agree)		Residents (agree)		Specialists (agree)		Total		*P* value
	*N*	%	*N*	%	*N*	%	*N*	%	
I understand that a solid background in the principles of genetics and biochemistry is a cornerstone of modern clinical practice	111	70.7	213	79.8	48	65.8	372	75.3	0.018
Understanding metabolism is essential to comprehend the biochemical changes manifested in cancer	113	71.5	225	84.3	57	79.2	395	79.9	0.007
I am acquainted with the most recent molecular advances in my field of specialty	70	44.6	159	60.7	34	47.9	263	54.8	0.003
I believe that enrollment in continuing medical education (CME) courses in the topics of biochemistry and genetics will positively reflect on the quality of health care provided to the patients	101	63.9	214	80.5	55	75.3	370	75.2	0.001
I am familiar with the most common molecular techniques applicable to my practice	97	61.4	175	65.8	45	62.5	317	64.0	0.636
I believe that biochemistry and genetics principles added a significant value to contemporary clinical practice	107	68.2	195	73.9	55	75.3	357	72.4	0.367
I am frequently exposed to research articles that discuss the molecular basis of diseases	61	38.9	116	44.1	22	32.8	199	41.3	0.203
Upon reading research articles, I have enough background to understand the molecular tools employed by the authors to support their conclusions	75	47.5	136	50.7	30	41.7	241	48.6	0.377

Data are presented as numbers and percentages.

**Table 5:** Attitudes and perceptions of Jordanian physicians toward the current level of knowledge on biochemistry and genetics among medical students.

Statement	Interns (agree)		Residents (agree)		Specialists (agree)		Total		*P* value
	*N*	%	*N*	%	*N*	%	*N*	%	
I believe that the current medical students are receiving more adequate training in biochemistry and genetics	62	39.2	116	43.6	36	49.3	214	43.3	0.343
I sense that there is a knowledge gap in biochemistry and genetics between previous and recent medical students	85	53.8	145	54.9	41	56.2	271	54.8	0.942
I strongly advise students to adopt a career path in biochemistry and genetics	94	59.5	188	70.9	46	65.7	328	66.9	0.054
I recommend that medical students get more in-depth training in biochemistry and genetics	99	63.1	194	73.8	48	68.6	341	70.0	0.068
The medical school curriculum currently offered in Jordanian universities is lacking in areas of biochemistry and genetics	95	60.1	174	65.4	46	63.9	315	63.6	0.549
Biochemistry and genetics were among my least favourite subjects during medical school	104	66.2	143	54.4	41	57.7	288	59.1	0.057
I believe that the main reason behind “phobia from Biochemistry” among students is that biochemistry depends heavily on abstract concepts that are intellectually challenging	97	61.8	184	69.2	57	80.3	338	68.9	0.019
The best way to motivate students to learn biochemistry is by translating biochemical concepts into relevant clinical scenarios	121	77.1	212	80.3	59	80.8	392	79.4	0.69
I support restructuring the curriculum in the Jordanian medical schools by integrating biochemistry and genetics with bedside teaching	111	71.2	189	71.6	50	68.5	350	71.0	0.874

Data are presented as numbers and percentages.

**Table 6:** Multivariate analysis of variables associated with the attitude of physicians toward the integration of biochemistry and genetics into clinical practice, advances in molecular medicine, the need for continuous education, and medical students' knowledge on biochemistry and genetics.

Variable	Attitude toward the integration of biochemistry and genetics concepts into clinical practice			Attitude toward contemporary advances in molecular medicine			Attitude toward the need for continuous education in biochemistry and genetics			Attitude toward medical students' knowledge on biochemistry and genetics		
	Mean	SD	*P* value	Mean	SD	*P* value	Mean	SD	*P* value	Mean	SD	*P* value
*Location*			0.799			0.364			0.310			0.306
Central	4.03	0.42		3.80	0.56		3.64	0.57		3.67	0.52	
Northern	3.96	0.49		3.79	0.60		3.87	0.61		3.76	0.52	
*Country of graduation*			0.219			0.644			0.838			0.730
Jordan	4.00	0.44		3.81	0.58		3.70	0.59		3.70	0.53	
Others	4.07	0.41		3.73	0.50		3.60	0.54		3.66	0.49	
*Job title*			0.206			0.170			0.101			0.725
Intern	3.94	0.38		3.75	0.57		3.58	0.52		3.64	0.52	
Resident	4.07	0.44		3.86	0.56		3.78	0.59		3.72	0.53	
Specialist	3.97	0.51		3.69	0.60		3.58	0.63		3.69	0.51	
*Employment sector*			0.002			0.049			0.000			0.479
Private	4.08	0.55		3.87	0.77		4.20	0.56		3.81	0.60	
Royal Medical Services	3.91	0.46		3.69	0.54		3.56	0.58		3.66	0.53	
Ministry of Health	4.06	0.40		3.85	0.54		3.67	0.54		3.68	0.51	
*Years of experience*			0.007			0.107			0.082			0.296
<5	3.98	0.41		3.79	0.58		3.69	0.58		3.67	0.53	
≥5	4.05	0.47		3.80	0.56		3.68	0.58		3.71	0.51	

Data are presented as mean and SD. *P* value was calculated using a general linear procedure.

## Data Availability

The datasets used and/or analysed during the current study are available from the corresponding author on reasonable request.
